# A Unique Case of Metastatic Cutaneous Squamous Cell Carcinoma Presenting Like Carcinoma en Cuirasse on the Face

**DOI:** 10.7759/cureus.40160

**Published:** 2023-06-08

**Authors:** Patrick Whitman, Stephanie Howerter, William Lear

**Affiliations:** 1 Dermatology, Silver Falls Dermatology, Salem, USA; 2 Dermatology, The Skin Surgery Center, Martinsville, USA; 3 Dermatology, Silver Falls Dermatology, Corvallis, USA

**Keywords:** moh's micrographic surgery, immune check-point inhibitor, cancer metastasis, chronic lymphocytic leukemia (cll), squamous cell carcinoma (scc)

## Abstract

Carcinoma en cuirasse (CeC) is a rare case of cutaneous metastases most commonly seen in the breast or visceral organs. The term carcinoma en cuirasse is largely used to describe the coalescing and fibrotic textural changes in the skin that can be seen in these metastatic lesions, which often manifest in a large plaque-like distribution. While most cases of CeC are found on the trunk, CeC has been reported in other body locations. However, to our knowledge, it has not yet been described on the face. In this report, we discuss a rare case of metastatic cutaneous squamous cell carcinoma (cSCC) that presented on the head and neck of a 67-year-old female, for which we have coined the term "carcinoma en bascinet." This novel term stems from the fibrotic changes associated with significant metastatic carcinomas of the head and neck, which bear a resemblance to a *bascinet*, which is a medieval-style helmet worn by European soldiers during the 14th and 15th centuries. We present this case of *carcinoma en bascinet* secondary to metastatic cSCC to demonstrate how metastatic cSCC can present in a facial distribution, causing significant morbidity and, as in this case, mortality. We hope that this case will increase the awareness of the highly variable presentation of metastatic cSCC, specifically as an extensive papulonodular and fibrotic plaque, allowing patients to receive early initiation of systemic therapy for symptom management and hence maximizing their quality of life.

## Introduction

Carcinoma en cuirasse (CeC) is a term initially coined by French anatomist Alfred Velpeau in his 1856 *Treatise on Diseases of the Breast and Mammary Region*, who observed that metastatic lesions from breast cancer would occasionally present in a manner resembling that of a medieval breastplate or cuirasse [1]. CeC is a rare presentation of cutaneous metastases most commonly seen in the breast or visceral organs. The term "carcinoma en cuirasse" is largely used to describe the coalescing and fibrotic textural changes in the skin that can be seen in these metastatic lesions, which often arrange themselves in a large plaque-like distribution [2]. While most cases of CeC are found on the trunk, CeC has been reported in other body locations. However, to our knowledge, it has not yet been described on the face. In this report, we discuss a rare case of metastatic cutaneous squamous cell carcinoma (cSCC) that presented on the head and neck of a 67-year-old female, for which we have coined the term “carcinoma en bascinet.” This novel term stems from the fibrotic changes associated with significant metastatic carcinomas of the head and neck, which bear a resemblance to a bascinet, which is a medieval-style helmet worn by European soldiers during the 14th and 15th centuries.

## Case presentation

A 67-year-old female presented with non-tender nodules on her scalp and forehead with some associated alopecia and focal cervical lymphadenopathy. Her medical history includes an eight-year duration of chronic lymphocytic leukemia (CLL) and non-melanoma skin cancer. The two sites were biopsied, and the pathology results were consistent with invasive cSCC, at which point she was referred for Mohs micrographic surgery (MMS). Upon presentation for MMS, she was noted to have a firm nodule on her scalp that was affixed to her underlying tissues, as well as persistent cervical and clavicular lymphadenopathy. Her forehead lesion was treated, resulting in MMS with complete clearance of the lesion. However, due to the persistence of her lymphadenopathy and the firm and fixed nature of the scalp lesion, she was referred to head and neck surgical oncology for further evaluation and treatment. A cervical lymph node biopsy was performed, and the pathology revealed nodal CLL. The scalp lesion was subsequently treated with wide local excision (WLE), and the pathology results revealed the presence of cSCC with perineural invasion and a positive margin, prompting adjuvant radiation therapy.

**Figure 1:**
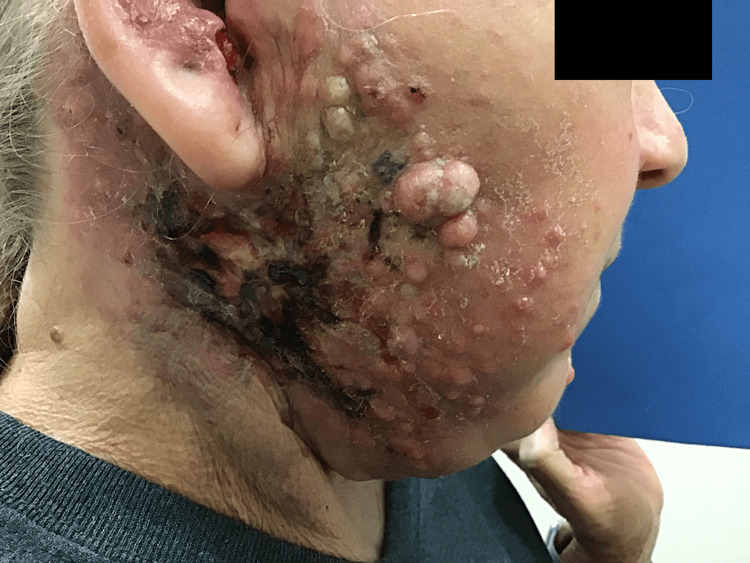
Carcinoma en bascinet presentation of poorly differentiated metastatic cSCC cSCC: cutaneous squamous cell carcinoma

**Figure 2:**
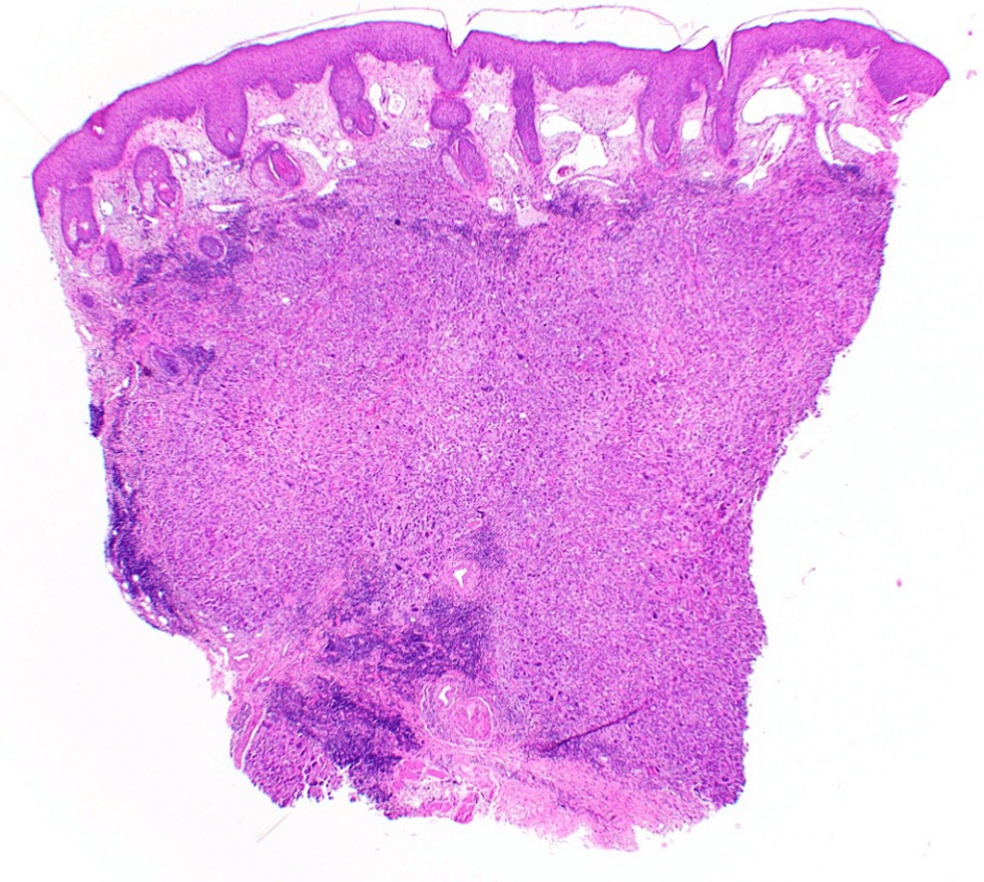
Punch biopsy at 4x magnification showing a dense dermal collection of disorganized atypical keratinocytes indicating poorly differentiated metastatic cSCC cSCC: cutaneous squamous cell carcinoma

At her six-month follow-up appointment, the patient was referred from oncology to dermatology for a rash consisting of papules and nodules on her right lateral cheek, ear, and neck (Figure 1). Clinically, these lesions were consistent with CeC, and two biopsies were performed. The pathology results were consistent with poorly differentiated metastatic cSCC (Figure 2).

Due to the extent of the metastases, the decision was made to initiate the patient on pembrolizumab. Unfortunately, due to the progression of her disease, this patient passed away within several months of her diagnosis.

## Discussion

CeC is an exceptionally rare presentation of metastatic carcinoma of the breast or other internal organs, and it is a significantly rarer variant of metastatic cSCC. A PubMed literature review revealed only two other case reports demonstrating CeC as a presentation of metastatic cSCC, one involving the pubic area from a penile cSCC and the other involving the trunk of an immunosuppressed patient with a history of renal transplant [3,4]. The case we present here appears to be the first reported case of CeC in a facial distribution, secondary to metastatic cSCC.

The cSCC metastasis in this case clinically appears to be in a V3 dermatomal distribution. This poses an interesting theory regarding a zosteriform pattern for the metastatic spread of cSCC. Unfortunately, due to the significant rarity of extensive metastatic cSCC, many more cases would be needed to confirm this theory.

According to a 2005 study, patients with CLL have a significantly increased risk of secondary malignancies, including metastatic cSCC, as well as increased mortality from metastatic cSCC [5]. This is believed to be secondary to a hindrance in immunosurveillance due to incompetent B-cells, T-cells, and natural killer cells, thereby allowing for higher-grade carcinomas to develop and grow more rapidly than in patients without CLL [6,7]. Additionally, the chronic lymphadenopathy that accompanies CLL can potentially cause a delay in the diagnosis of secondary metastatic malignancy from the assumption that any lymphadenopathy is attributed to CLL. Recommendations for patients with CLL include early and frequent biopsies of suspicious skin lesions, followed by aggressive and early treatment. The current guidelines of the American Academy of Dermatology recommend treatment of metastatic unresectable cSCC with chemotherapeutic agents, such as cisplatin, 5-fluorouracil, or cetuximab, or immune checkpoint inhibitors, such as pembrolizumab, with or without adjuvant radiation therapy, and close follow-up by a multidisciplinary team [8].

## Conclusions

We present this case of carcinoma en bascinet secondary to metastatic cSCC to demonstrate how metastatic cSCC can present as a facial distribution, causing significant morbidity and, as in this case, mortality. We hope that this case will increase awareness of the highly variable presentation of metastatic cSCC, specifically as an extensive papulonodular and fibrotic plaque, allowing patients to receive early initiation of systemic therapy for symptom management and thus maximizing their quality of life.

## References

[1] Velpeau A (1856). A Treatise of the Diseases of the Breast and Mammary Region. http://344.

[2] Varghese A, Singh A, Ambujam S (2013). Carcinoma en cuirasse: A cutaneous clue for systemic malignancy. International Journal of Preventative Medicine.

[3] Brady KL, Scott GA, Gilmore ES (2015). Cutaneous metastasis from penile squamous cell carcinoma resembling carcinoma en cuirasse. Dermatol Online J.

[4] Lorentzen H (2008). Carcinoma “en cuirasse” from cutaneous squamous cell carcinoma in a renal transplant patient. Transplantation.

[5] Mehrany K, Weenig RH, Lee KK, Pittelkow MR, Otley CC (2005). Increased metastasis and mortality from cutaneous squamous cell carcinoma in patients with chronic lymphocytic leukemia. J Am Acad Dermatol.

[6] Wong J, Breen D, Balogh J, Czarnota GJ, Kamra J, Barnes EA (2008). Treating recurrent cases of squamous cell carcinoma with radiotherapy. Curr Oncol.

[7] Cheson BD (2001). The chronic lymphocytic leukemias. Cancer, Principles and Practice of Oncology.

[8] Kim JY, Kozlow JH, Mittal B, Moyer J, Olenecki T, Rodgers P (2018). Guidelines of care for the management of cutaneous squamous cell carcinoma. J Am Acad Dermatol.

